# Are Ceramic Cages an Option for Lumbar Interbody Fusion? A Comparative Biomechanical Numerical Study

**DOI:** 10.1002/jsp2.70215

**Published:** 2026-09-01

**Authors:** Davide Ninarello, Luigi La Barbera

**Affiliations:** ^1^ Laboratory of Biological Structure Mechanics, Department of Chemistry, Materials and Chemical Engineering “Giulio Natta” Politecnico di Milano Milan Italy

**Keywords:** bioceramic, biomechanics, cage, finite element model, lumbar interbody fusion

## Abstract

**Background:**

The treatment of severe disc pathologies through lumbar interbody fusion (LIF) involves the insertion of an interbody cage into the degenerated disc space, together with a supporting fixation system. Conventional cage materials, such as Ti6Al4V and PEEK, present several limitations, most notably their non‐biodegradability, increasing the risk of cage migration and long‐term subsidence (up to 40% incidence). Bioceramics may represent an option while addressing the issue of biodegradability. This study preliminarily investigates the applicability of a commercial bioceramic as an interbody cage material for LIF.

**Methods:**

First, an intact L4‐L5 functional spine unit (FSU) was validated under both pure moments and combined loading conditions. Subsequently, all surgical steps of the LIF procedure were simulated, followed by postoperative physiological loading to compare the mechanical performance of the ceramic device with standard titanium cages. Two surgical techniques, posterior‐LIF (PLIF) and eXtreme‐LIF (XLIF), were analyzed. Each simulation assessed the mechanical strength of the interbody cage, the posterior fixation, and the vertebral bone, together with the FSU kinematic.

**Results:**

The intact model accurately predicted literature‐reported kinematics and intradiscal pressure, and the LIF model reproduced forces on titanium cages consistent with in vitro measurements on human specimens. Both XLIF and PLIF procedures resulted in significant kinematic stabilization of the FSU, with > 70% reduction in range of motion. For PLIF, ceramic cages sustained applied loads with no predicted failure, while XLIF showed minimal interface failure (< 0.6%). Compared with titanium, ceramic cages significantly reduced adjacent vertebral strain, lowering the subsidence risk, without overloading the posterior fixation system.

**Conclusion:**

The present study supports the potential of bioceramics as viable materials for interbody cage applications in LIF procedures. Experimental tests and clinical validation should be accompanied to further cage design optimization to enhance the safety margins of this novel bioceramic device and ensure proper clinical effectiveness.

## 1 Introduction

Low back pain (LBP), which affects almost 1 in 13 people [1], is originated by several factors, from interbody disk degeneration up to fractures and tumors. Typical LBP non‐surgical treatments involve corrective gymnastics; however, for critical conditions, surgery is required. In this scenario, lumbar interbody fusion (LIF) is the gold standard for the treatment of these pathologies. LIF consists in the implantation of an interbody cage within the intervertebral space to allow the fusion of two adjacent vertebrae. Commonly used alongside the interbody cage is a posterior fixation system involving pedicle screws and rods.

A number of LIF techniques have been developed over the years, which are differentiated by the access point to the intervertebral space. These methods range from posterior access (PLIF) to anterior approach (ALIF), with many intermediate methods (TLIF, LLIF, OLIF, etc.) in between [2]. The shape of the cage is determined by the technique used. For instance, the PLIF involves the posterior insertion of two small cages, whereas the LLIF/XLIF involves the lateral implantation of a single large cage [3]. Cage's shape, size, material and positioning strongly affect the postoperative spinal biomechanics, influencing the effectiveness and success of the surgery [4, 5, 6]. Standard materials such as titanium alloys (Ti6Al4V) and polyetheretherketone (PEEK) are widely used thanks to their main properties as excellent strength and good osteointegrative properties for the former, and bone‐like mechanical properties and radiolucency for the latter. However, both materials present some limitations: Ti alloys present high rates of subsidence, with up to 42% incidence rates [7, 8] while the hydrophobicity of PEEK can lead to delayed bone growth, potentially leading to pseudoarthrosis and need for revision surgery [9]. Some cages address these challenges by combining the benefits of both materials, exploiting a PEEK core with a titanium coating to enhance osteointegration [10].

However, both Ti alloys and PEEK shared the same disadvantage to be non‐resorbable. It has been observed long‐term issues due to cage mobilization and subsidence after several months from the surgery [11, 12]. To overcome this major limitation biodegradable materials can be exploited as alternative to standard approaches [13, 14]. Up to date, three biodegradable interbody cages are present on the market: Hydrosorb a 70:30 poly(l‐d, l‐lactide) polymer (Medtronic Sofamor Danek, Memphis, Tennessee, USA) [15], Smartbone a combined bovine mineral bone matrix with resorbable polymers and collagen fragments (Industrie Biomediche Insubri SA—I.B.I., Mezzovico‐Vira, Switzerland) and Bio‐AVS, a bone allograft plug obtained from human femur and tibia (Stryker Corporation, Kalamazoo, Michigan, USA). Regardless the CE mark and FDA approval, recent studies showed bad long‐term outcomes for Hydrosorb with several non‐unions cases and high osteolysis rates (45%), due to the degradation products of the polymer [16]. Regarding Smartbone and Bio‐AVS plugs, no clinical studies have been found, precluding a thorough discussion on their performances.

In recent years, synthetic biphasic ceramics have attracted growing interest as bone substitutes for the treatment of defects in load‐bearing regions [17]. To the authors' knowledge, a single study by Hojo et al. [18] tried to verify the applicability of synthetic ceramic interbody cage for lumbar fusion, but no recent literature is available. To address this gap, the present study evaluates the feasibility of brittle bioceramic XLIF and PLIF cages by investigating their primary mechanical stability and their biomechanical effects on the adjacent vertebrae compared to conventional titanium cages.

## 2 Materials and Methods

### 2.1 Intact Spine Model

A previously validated fully parametric thoraco‐lumbar spine finite element model [19] was here exploited focusing on the L4‐L5 functional spine unit (FSU). The FSU is composed by two vertebral body, divided into cortical and trabecular bone, posterior processes, seven 3D solid ligaments (anterior and posterior longitudinal ligaments, flavum ligament, interspinous ligament, capsular ligament, intertransverse ligament and supraspinous ligament), the intervertebral disk and the facet joints. Trabecular and cortical bone were simulated as elastic transversely isotropic [20], the vertebral arch as isotropic linear elastic, while the mechanical behavior of each intervertebral soft tissue was obtained through a backward stepwise calibration [19]. The seven spinal ligaments were represented as hyperelastic isotropic (Ogden material model), the anulus fibrosus was divided into four quadrants and represented through a hyperelastic anisotropic Holzapfel–Gasser–Ogden material model, while the nucleus pulposus was simulated as a fluid cavity. All materials parameters are resumed in Table 1. The vertebral bodies were meshed with tetrahedral elements as the vertebral arch while the ligaments were meshed with hexahedron elements, mesh sizes were defined after a proper convergence analysis, similarly to [19] where the L4‐L5 FSU was extracted. All simulations were carried out with ABAQUS 2022 software (Dassault Systèmes, Simulia Corp., USA). The intact model was validated considering simplified loads (pure moment) and complex load (combination of pure moment and follower load), to represent standing (STD), flexion (FLEX), extension (EXT), axial rotation (AR), and lateral bending (LB) [21], as summarized in Table 2.

**TABLE 1:** Material properties of all anatomical structures of the model.

Anatomical structure	Constitutive model	Constitutive parameters		References
Cortical bone	Linear elastic	*E*_11_ = *E*_33_ = 11.3 GPa *E*_22_ = 22 GPa *G*_12_ = *G*_23_ = 5.4 GPa *G*_13_ = 3.8 GPa	*ν*_12_ = *ν*_23_ = 0.2 *ν*_13_ = 0.48	[20]
Trabecular bone	Linear elastic	*E*_11_ = *E*_33_ = 140 MPa *E*_22_ = 200 MPa *G*_12_ = *G*_23_ = 48.3 MPa *G*_13_ = 48.3 MPa	*ν*_12_ = *ν*_23_ = 0.32 *ν*_13_ = 0.45	[20]
Vertebral arch	Linear elastic	*E* = 6 GPa	*ν* = 0.3	[19]
Annulus fibrosus	Holzapfel–Gasser–Ogden			
Lateral quadrants		*C*_10_ = 2.5 MPa, *D* = 2.45 MPa^−1^, *k*_1_ = 1.08 MPa, *k*_2_ = 50, *k* = 0.25, 2 fiber families, angle = ±33.8°		[19]
Internal anterior/posterior quadrants		*C*_10_ = 1 MPa, *D* = 0.71 MPa^−1^, *k*_1_ = 1.08 MPa, *k*_2_ = 50, *k* = 0.25, 2 fiber families, angle = ±33.8°		[19]
External anterior/posterior quadrants		*C*_10_ = 0.36 MPa, *D* = 0.3 MPa^−1^, *k*_1_ = 1.08 MPa, *k*_2_ = 90, *k* = 0.33, 2 fiber families, angle = ±33.8°		[19]
Nucleus pulposus		Fluid cavity		[19]
Anterior longitudinal ligament	Ogden (*n* = 1)	*μ* = 3.2 MPa, *α* = 62.5, *D* = 0.088 MPa^−1^		[19]
Posterior longitudinal ligament	Ogden (*n* = 1)	*μ* = 10.8 MPa, *α* = 6.2, *D* = 0.362 MPa^−1^		[19]
Flavum ligament	Ogden (*n* = 1)	*μ* = 0.99 MPa, *α* = 1.8, *D* = 18.1 MPa^−1^		[19]
Interspinous ligament	Ogden (*n* = 1)	*μ* = 0.99 MPa, *α* = 1.8, *D* = 18.1 MPa^−1^		[19]
Capsular ligament	Ogden (*n* = 1)	*μ* = 0.99 MPa, *α* = 10.0, *D* = 10.0 MPa^−1^		[19]
Intertransverse ligament	Ogden (*n* = 1)	*μ* = 1.2 MPa, *α* = 35.2, *D* = 0.762 MPa^−1^		[19]
Supraspinous ligament	Ogden (*n* = 1)	*μ* = 0.99 MPa, *α* = 24.0, *D* = 1.07 MPa^−1^		[19]

*Note:* Local reference system (1–2–3) for cortical and trabecular bone is illustrated in Figure 2a.

**TABLE 2:** Pure moment and combined pure moment and follower load representative of standing, flexion, extension, axial rotation, and lateral bending [21].

	Pure moment (Nm)	Follower load (N)
Pure moment		
Flexion	7.5	—
Extension	7.5	—
Axial rotation	5.0	—
Lateral bending	7.5	—
Combined loading		
Standing	—	500
Flexion	7.5	1175
Extension	7.5	500
Axial rotation	5.5	720
Lateral bending	7.8	700

### 2.2 Lumbar Interbody Fusion Models

**FIGURE 1:**
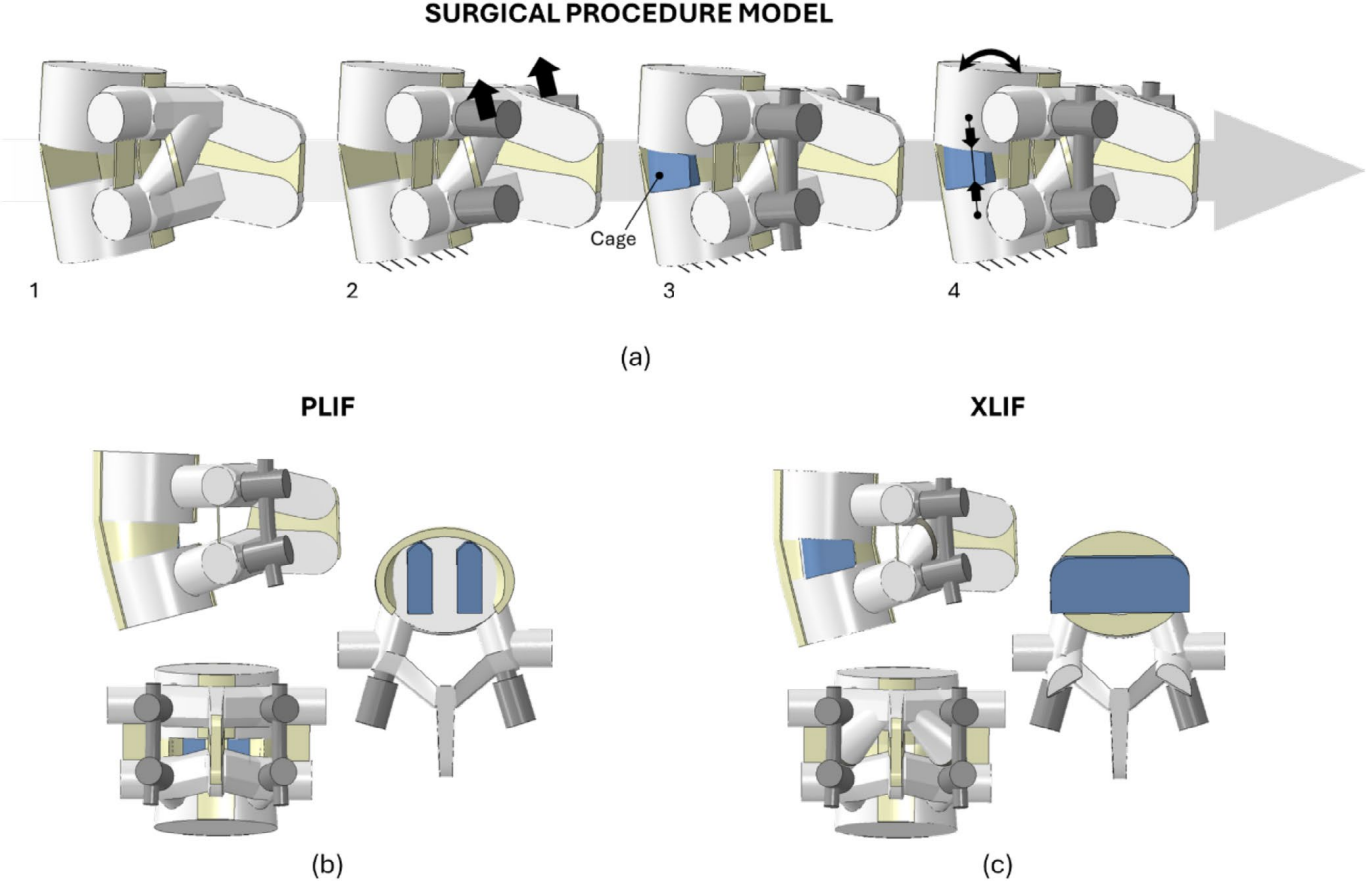
(a) Simulation of surgical procedure of LLIF and functional load: (1) disc preparation, (2) vertebral distraction, (3) cage insertion and posterior fixation, and (4) functional loading. (b) PLIF numerical model and (c) XLIF numerical model. The removal of specific osteo‐ligamentous tissues, according to each surgical technique, can be appreciated.

Two standard LIF techniques were simulated: a lateral‐LIF (XLIF) and a posterior‐LIF (PLIF). The surgery was represented through a four‐step simulation (Figure 1a):

1. Removal of the spinal structures to allow the implantation of the interbody cage
  - *PLIF*: Bilateral facetectomy, partial removal of the posterior longitudinal ligament, partial posterior discectomy, and nucleotomy (Figure 1b)
  - *XLIF*: Partial lateral discectomy and nucleotomy (Figure 1c)
2. Distraction of the vertebrae, applying a displacement in cranial direction to the head of the L4 titanium pedicle screws (*E* = 110 GPa and *ν* = 0.3) sufficient to avoid interference between the vertebra and the cage
3. Insertion of the cage(s), ensuring contact with the inferior endplate, and release of distraction, ensuring contact with the superior endplate (friction coefficient equal to 0.8 [22, 23]). Fixation of the L4‐L5 FSU through titanium rods (diameter 5.5 mm; *E* = 110 GPa and *ν* = 0.3)
4. Functional loadings including both pure moments and complex functional loadings (Table 2)

**FIGURE 2:**
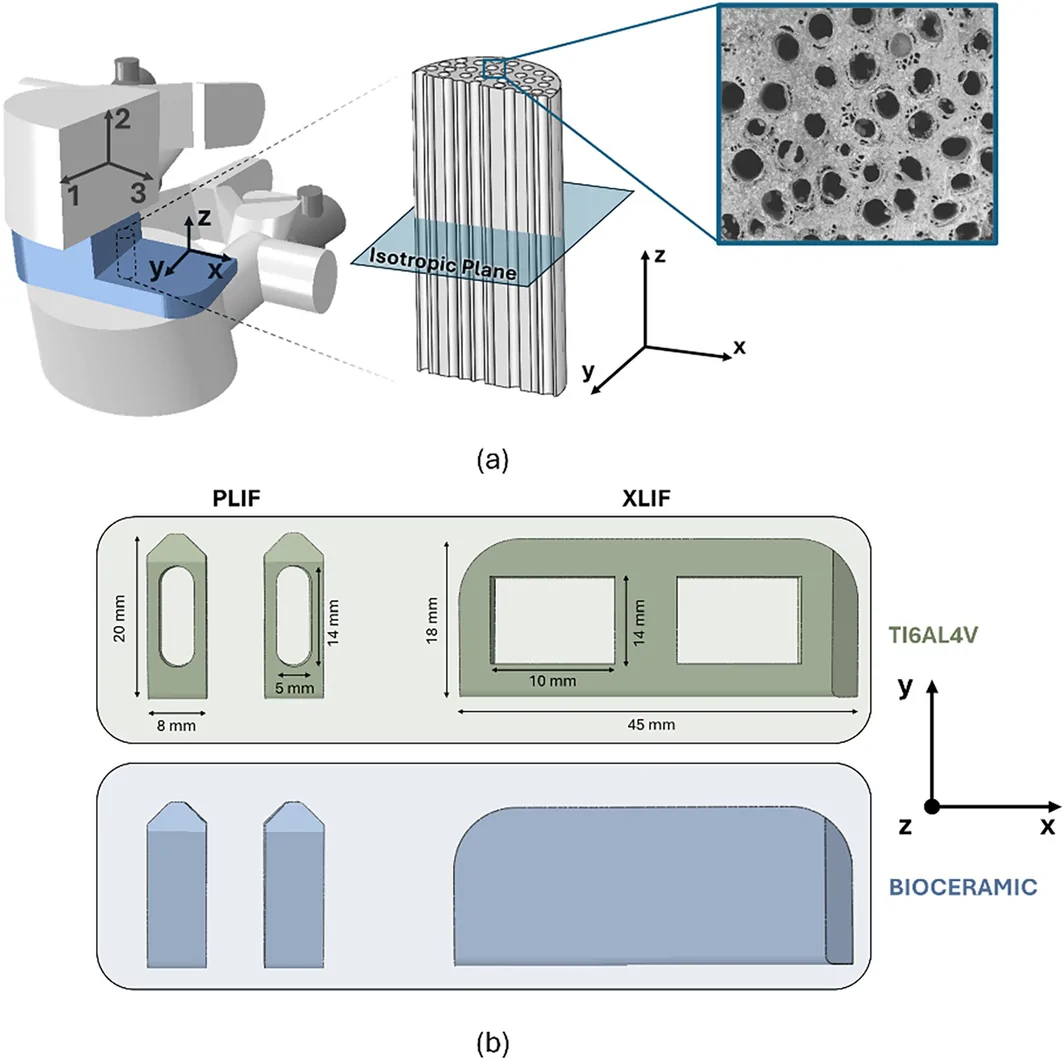
(a) Representation of b.Bone and its local reference system (*x*–*y*–*z*) as material for the interbody cage. Local bone reference system (1–2–3) is also represented. (b) Design and dimension of both PLIF and XLIF interbody cages. Unlike the titanium cages (in green), the bioceramic cages (in blue) did not need internal holes to host the bone graft due its high porosity.

For each technique, two materials were simulated. First, standard Ti6Al4V (*E* = 110 GPa and *ν* = 0.3), then a brittle ceramic material. For the latter case, it was considered b.Bone (Greenbone Ortho S.p.A., Faenza, Italy), a porous (58.20% ± 3.56%) biphasic ceramic obtained from rattan wood through a biomorphic transformation [24]. The material has been previously thoroughly characterized [25] and a transversely isotropic model was calibrated (Figure 2a), as briefly summarized in Supporting Information A. The final material compliance matrix (***D***) was defined as follows:

$$D=\left[\begin{array}{cccccc} 0.63 & -0.17 & -0.06 & 0 & 0 & 0\\ -0.17 & 0.63 & -0.06 & 0 & 0 & 0\\ -0.06 & -0.06 & 0.22 & 0 & 0 & 0\\ 0 & 0 & 0 & 1.59 & 0 & 0\\ 0 & 0 & 0 & 0 & 0.72 & 0\\ 0 & 0 & 0 & 0 & 0 & 0.72 \end{array}\right]\;\mathrm{GP}a^{-1}$$

The XLIF cage presented a 45 × 18 mm external footprint, but the titanium one had two holes to host bone graft (Figure 1b). For the PLIF case, the footprint was reduced to 8 × 20 mm (for each cage), and also in this case the titanium cages presented a hole for bone graft insertion (Figure 2b). While the same element types as in the intact model were used for the spinal structures, the mesh size of the endplate in contact with the cage(s) was refined. In particular, an average element size of 0.3 mm was applied at the interface between cage(s) and bone following a mesh sensitivity analysis based on local stresses and strains. Finally, the interbody cage(s) and the posterior instrumentation were meshed with hexahedral elements, with mesh size derived from previous sensitivity analyses [26, 27].

Model credibility was assessed replicating the experimental test described in the work by Calek et al. [28], simulating step‐by‐step the surgery as described above up to the vertebral release phase. After vertebral release, a compression force was applied to the screw heads as defined in [28], simulating the tightening of the posterior fixation.

### 2.3 Data Analysis

#### 2.3.1 Intact Model

The intact model was validated in terms of range of motion (RoM) and intradiscal pressure (IDP) compared with in vitro tests on human spine [29] and numerical models [21], respectively.

#### 2.3.2 LIF Model

First, the credibility of the instrumented LIF models was ensured by comparing both the force acting on the cages [28] and the postoperative percentage reduction in the RoM with in vitro tests on human specimens [29, 30, 31].

The mechanical strength of standard titanium cages and titanium rods was assessed using a safety factor (SF), calculated as the ratio between the material's yield strength (885 MPa) and the peak von Mises stress calculated in the simulation.

Instead, the mechanical strength of the bioceramic interbody cage(s) and vertebral bone was evaluated exploiting a strain‐based failure criteria [32], by comparing the strain values computed within each material with the corresponding ultimate strain limits. Specifically, for ceramic cages the material's transverse isotropy was accounted for by evaluating the strains along the axes of the cage local reference system (Figure 2a). To quantify potential failure risk within the ceramic cage, the percentage of the cage volume exceeding the established strain limits (0.43% in axial compression, 0.27% in transverse compression, see Supporting Information A) was determined for each simulation. While bone resistance at the cage‐bone interface was evaluated by comparing bone maximum and minimum principal strains to their ultimate strains (2% in compression and 1.6% in tension) [33]. This comparison yielded the bone failed surface, calculated as the ratio between the failed surface area and the initial cage‐bone contact surface.

## 3 Results

### 3.1 Model Validation and Credibility

#### 3.1.1 Intact Mode

**FIGURE 3:**
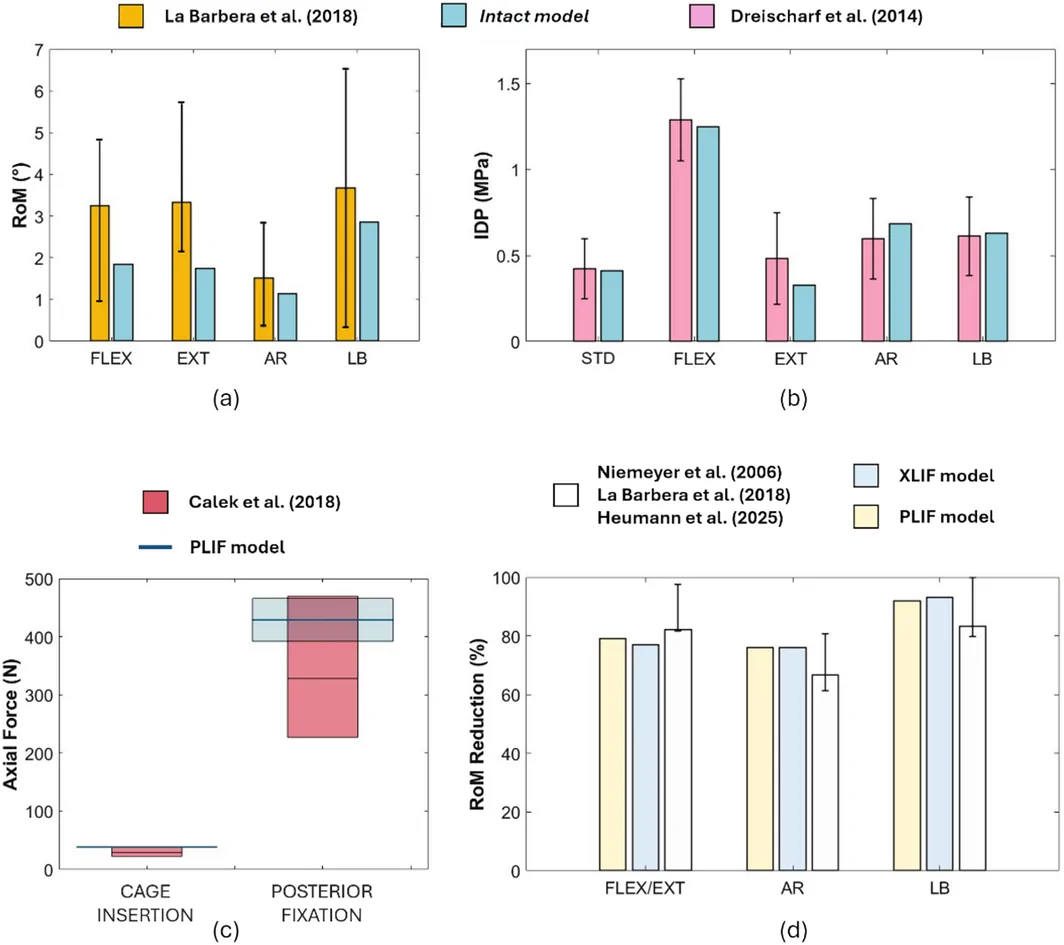
Validation of the intact model in terms of RoM under pure moments (a) and IDP under pure compression (standing position) and complex loadings (b) between the intact spine model and, respectively, in vitro [29] and in silico [21] analysis. Credibility assessment of the instrumented LIF models in terms of forces acting on the cages (c) compared with in vitro tests and in terms of RoM reduction (d) compared with different literature in vitro tests on human spine specimens.

Both in terms of RoM (°) and IDP (MPa), the numerical model correctly predicted results in line with literature [21, 29] as shown in Figure 3a,b.

#### 3.1.2 LIF Model

The model predicted a compressive force on the titanium PLIF cages equal to 38.7 N and 428.9 N (392.4–465.5 N) respectively after the cages insertion and the posterior fixation. These results are in line with the in vitro tests performed by Calek et al. [28] on human instrumented L4‐L5 FSU with PLIF cages (Figure 3c). Furthermore, the LIF model correctly predicted the percentage reduction in RoM as observed by several authors with in vitro tests on instrumented human spines [29, 30, 31], with a mean reduction of 78%, 76%, and 92% for, respectively, flexion/extension, axial rotation, and lateral bending (Figure 3d).

### 3.2 Mechanical Strength of Instrumentation and Vertebral Bone

#### 3.2.1 Interbody Cage

**FIGURE 4:**
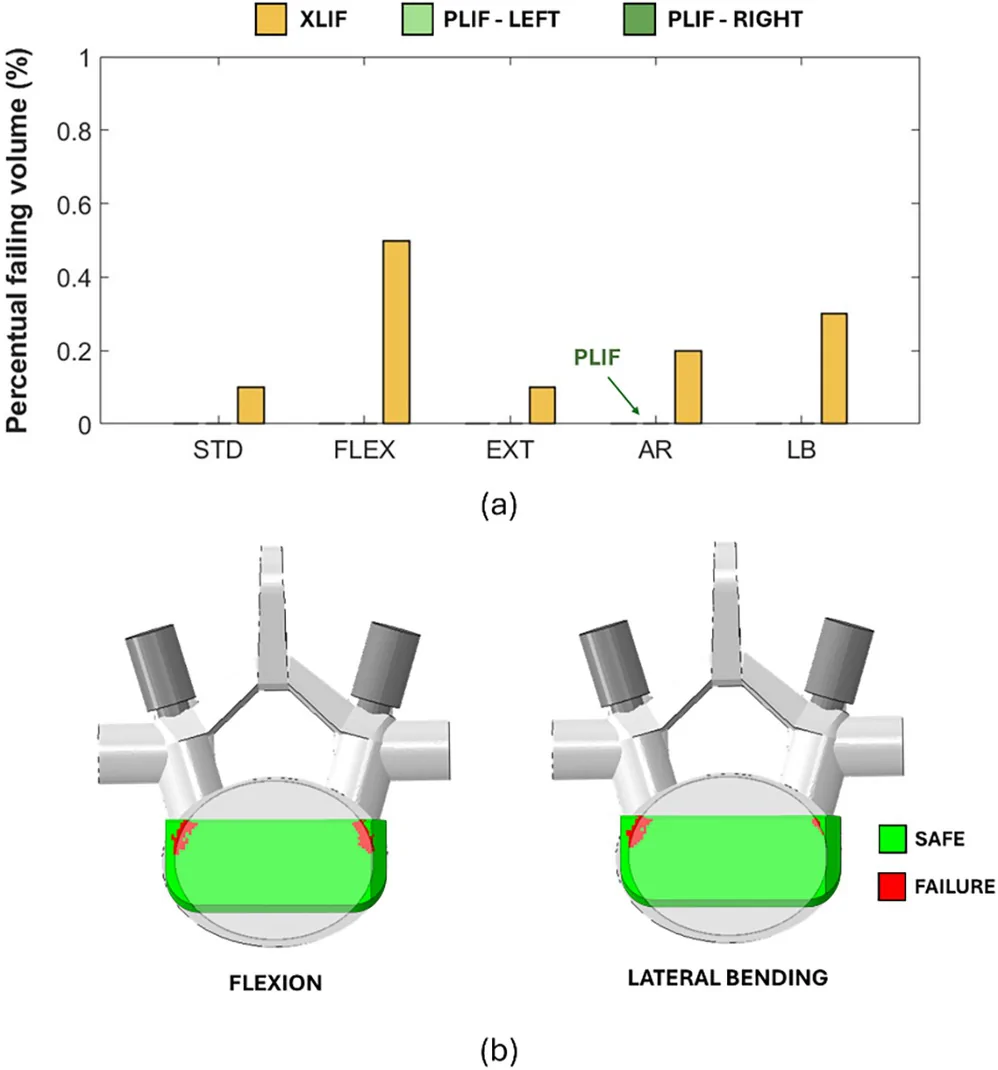
(a) Percentual cage failed volumes for ceramic XLIF and PLIFs in standing (STD), flexion (FLEX), extension (EXT), axial rotation (AR), and lateral bending (LB), and (b) for the XLIF cage in flexion and lateral bending is represented in red the region failed and in green the region which is able to withstand the load.

For all movements and techniques, titanium cages resulted in safe conditions. In particular, SF ranged between 1.9 (LB) and 2.6 (FLEX) for XLIF and between 25.0 (FLEX) and 95.1 (EXT) for PLIF. Regarding the ceramic material, no volume failed for the PLIF cages, while < 0.6% for the XLIF cage, with flexion the most critical condition (Figure 4a). The cage failing volumes were concentrated in the region of contact between the XLIF cage and the cortical shell (Figure 4b).

#### 3.2.2 Spinal Rods

For all movements and interbody cage materials, rods SF was > 1, confirming the instrumentation mechanical resistance. For PLIF, lateral bending represented the most critical condition, with the lowest SFs (SF = 13.7 and 13.8 for titanium and ceramic, respectively) followed by axial rotation (SF = 20.5 and 20.3), flexion (SF = 33.4 and 33.9), and extension (SF = 59.9 and 57.7), while for XLIF the lowest SF were observed for flexion (SF = 13.3 and 16.2 for titanium and ceramic respectively), followed by lateral bending (SF = 18.4 and 19.6), axial rotation (SF = 15.6 and 16.8), and extension (SF = 41.1 and 54.0). No significant difference was observed comparing titanium and ceramic cages, although a slight increase in rods' SF was observed for the ceramic XLIF compared to the titanium counterpart.

#### 3.2.3 Vertebral Bone

**FIGURE 5:**
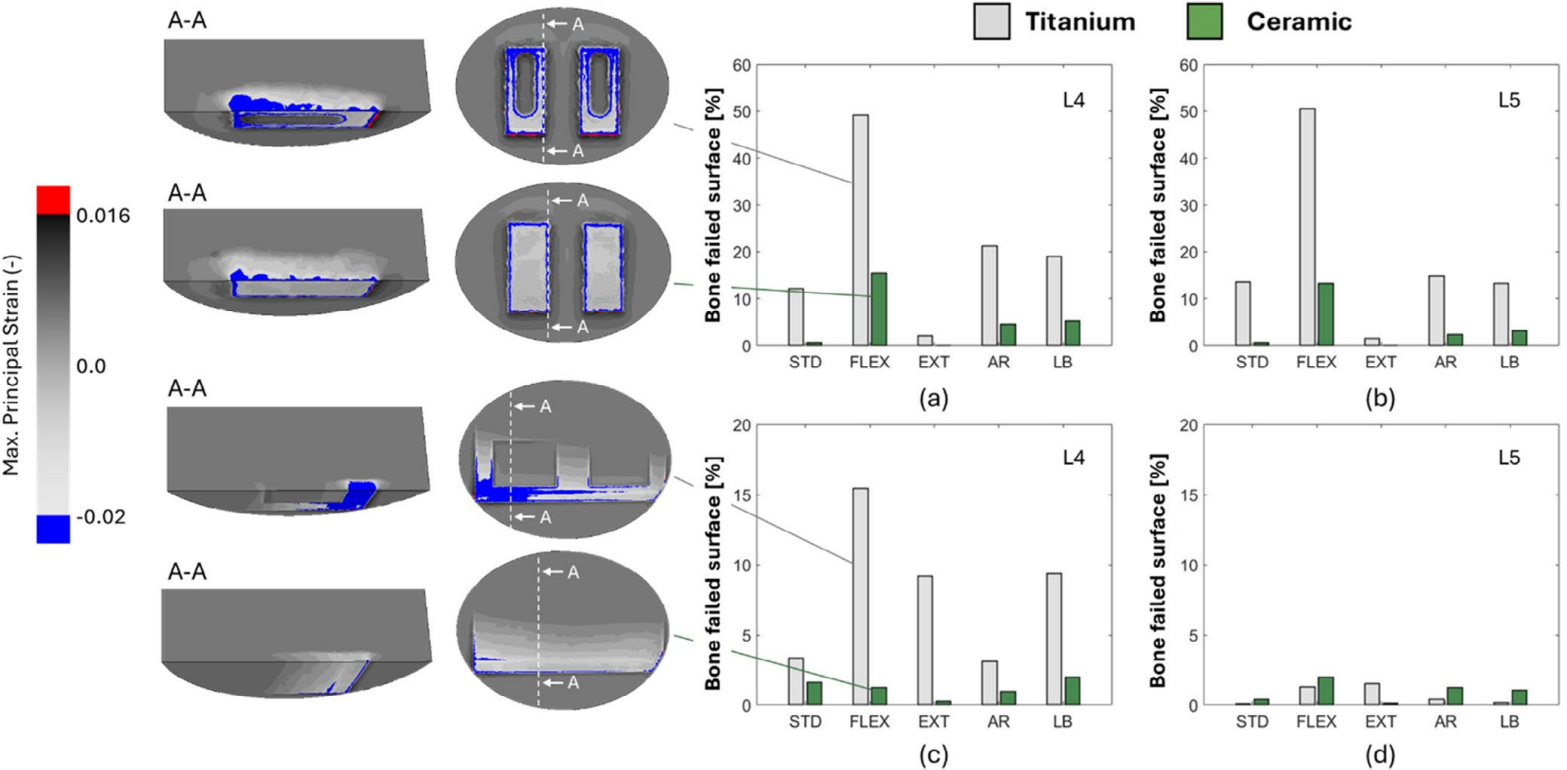
Bone failed surface (%) for PLIF at L4 (a) and L5 (b) and for XLIF at L4 (c) and L5 (d) in standing (STD), flexion (FLEX), extension (EXT), axial rotation (AR), and lateral bending (LB). Results for the titanium cages are presented in gray, while the ceramic cages are in green. The color maps (on the left) depict the maximum principal strain for the most critical loading condition (flexion) for both PLIF and XLIF, highlighting in red and blue the regions overcoming the tensile and compressive strain limits of bone, respectively; the cross‐section (A‐A) of the vertebra highlights the depth of failed bone.

With a PLIF approach, flexion was the most critical loading condition in terms of vertebral strength, regardless of the interbody cage material. When titanium cages were simulated, failure was predicted over 49.1% and 50.5% of the cage–bone contact surface at L4 and L5, respectively (Figure 5a,b). In contrast, ceramic cages exhibited substantially lower failed surfaces, limited to 15.4% at L4 and 13.2% at L5. The XLIF procedure yielded consistently lower bone failed surface values, which remained below 20% across all conditions (Figure 5c,d).

## 4 Discussion

### 4.1 Parametric Spine Model

In the present work, a fully parametric thoraco‐lumbar spine model generator [19] was used to obtain the L4–L5 functional spinal unit (FSU). In recent years, the use of parametric spine models has gained increasing interest as an alternative to conventional CT‐based segmented models. While CT‐based models are representative of a single patient‐specific morphology, CAD‐based parametric models can reproduce a wider range of anatomical configurations, enabling broader investigations while maintaining sufficient reliability and generalizability when compared with CT‐based counterparts [34, 35, 36]. Moreover, CAD‐based models do not require a complete CT dataset; instead, a limited set of patient‐specific morphological parameters is sufficient to generate a representative model, resulting in faster model preparation.

### 4.2 Model Validation and Credibility

Once the intact model was validated through comparison with literature data (Figure 3a,b), the present work focused on realistically simulating the LIF procedure, from vertebral distraction to posterior fixation and subsequent functional loading (Figure 1a). The model also accounted for friction at the cage–bone interface, which is totally neglected in several previous works [37, 38, 39, 40]. The credibility of the LIF model predictions was ensured by comparing the loads acting on the titanium cage and the postoperative RoM with in vitro measurements obtained from human specimens, showing good agreement with published data [29, 30, 31] (Figure 3c,d). These results support the credibility of the computational model built herein as a fundamental step for building confidence in the results generated by subsequent analyses.

### 4.3 Kinematic Stabilization

Both PLIF and XLIF surgery ensured effective stabilization of the L4‐L5 FSU with a percentage reduction in the RoM higher than 87%, in line with previous literature works [41, 42, 43]. Kinematic stabilization is primarily attributed to the presence of the posterior fixation system. Neither the surgical technique (PLIF vs. XLIF) nor the cage material (titanium vs. ceramic) had a significant effect on the RoM of the postsurgical model.

### 4.4 Rod Strength

Regardless of thefixator's key role in withstanding the external load, its safety was proved under all loading conditions for both interbody cage materials, obtaining values in line with previous studies [44, 45].

Furthermore, no significant difference in terms of rods' SF was observed between titanium and ceramic cage, similar to what wasobserved in [46] between Titanium and PEEK (which present an elastic modulus comparable to the bioceramic) PLIF cages. In the present work, it was noted an increase in the SF passing from XLIF to PLIF, which is in contrast with the results of Oikawa et al. [47], where slightly higher SFs were obtained considering LLIF surgery. However, it is worth noting that [47] differs from the present work in terms of interbody cage's shape, size, and positioning, boundary conditions and ligamentous structures removed, which strongly affect the load sharing between anterior and posterior column. Additionally, Oikawa et al. [47] assumed a bonded interface between the bone and the cage, unlike the frictional contact modeled here.

### 4.5 Titanium Cage

As for the rods, also titanium interbody cages presented SF higher than 1 for all simulated movements, ensuring sufficient mechanical resistance. Similar results were found by Fan et al. [45] and Yan et al. [48] for titanium PLIF cages with small discrepancies mostly due to the description of the bone–cage interface. While [45, 48] applied a bounded interface between bone and interbody cage, in the present work the frictional contact determined different forces exchange at the bone–cage interface.

### 4.6 Ceramic Cage

Since the considered ceramic material presented an inorganic bone‐like composition (biphasic calcium phosphate), but also a structure resembling human bone, it adopted the same strain‐based failure criteria as trabecular bone. The model predicted that the PLIF cages were able to withstand typical functional loadings, while the XLIF cages experienced minimal failing volumes (< 0.6%). This difference is mainly associated with the direct contact between the XLIF cage and the cortical shell of the adjacent vertebrae (Figure 4b). The pronounced mechanical mismatch between the bioceramic material (*E* = 4.5 GPa) and the cortical shell (*E*_11_ = *E*_33_ = 11.3 GPa and *E*_22_ = 22 GPa) resulted in fracture of the superficial portions of the cage. Nevertheless, the damaged regions were largely confined to the surface and did not reach the cage core. Based on these findings, a single lateral LIF with a smaller footprint limited to the trabecular bone could be advantageous to avoid cage failure.

In this study, the ceramic cage, due to its intrinsic porosity, was modeled without the holes commonly incorporated in standard interbody cages for bone graft placement and promotion of bony bridging (Figure 2b). Although ceramic cages can function as self‐grafting devices and therefore do not strictly require such openings, the inclusion of appropriately positioned holes could accelerate cage degradation and be tailored to balance bone formation and material resorption. In addition, transverse holes may be required to facilitate surgical handling and insertion.

### 4.7 Vertebral Bone

Two main factors affected the mechanical resistance of vertebral bone: LIF technique and interbody cage material. Regardless of the cage material simulated, the larger footprint of the XLIF cage led to a significant reduction in bone failure compared to the PLIF counterpart (Figure 5), in line with experimental findings [49]. Instead, for the same surgical technique, softer material (i.e., bioceramic) led to a reduction in bone failure compared to stiffer material (i.e., titanium alloy), due to lower mechanical mismatch with trabecular bone [50, 51].

### 4.8 Limitations

This work acknowledges certain limitations. First, the applied physiological loading conditions may not entirely capture the altered biomechanics observed in postoperative pathological patients. However, under the hypothesis of a complete restoration of correct spinal alignment following surgery, these loadings could be deemed reasonable.

Furthermore, this study only examined the mechanical resistance of the cage in the immediate postsurgical condition, consistent with previous studies in the literature [38, 52, 53]. Moreover, it assumed mechanical properties as measured in dry experiments [25], as in the short‐term the blood is expected to insufficiently permeate the cage material. While this approach is appropriate for assessing primary implant stability, it does not account for time‐dependent behaviors, such as fracture propagation and ceramic degradation. Unlike permanent implants, bioceramic cages are subjected to progressive degradation and replacement by newly formed bone, making their mechanical performance inherently transient. Future work should therefore focus on applying fracture propagation models and mechanobiological numerical models integrating cage degradation, enabling the prediction of time‐dependent behaviors and safety of the bioceramic interbody cage. Some simplifications were incorporated into the LIF numerical model. First, the contact between the vertebral endplate and the cage was simplified avoiding imperfect contacts and local indentations of the cage [38, 39, 40, 54, 55, 56, 57]. Pedicle screw insertion and rod‐screw connection were simulated using an embedded condition, thus neglecting the screw‐bone and rod‐screw interfaces. Furthermore, the vertebra was modeled with homogeneous materials and a thin cortical shell, whereas in reality, bone density may have a more heterogeneous spatial distribution. The friction coefficient between bioceramic material and bone was estimated from literature [22, 23], considering rough surface contact conditions. However, future experimental tests may allow a more accurate determination of this parameter. Finally, the mechanical properties assigned to bone and ligaments were based on material properties calibrated on healthy individuals, while patients undergoing LIF may present pathological conditions. Nevertheless, these simplifications are expected to have a minimal impact on the predicted mechanical resistance of the novel ceramic interbody cage, thereby allowing for a reliable assessment of its applicability for LIF procedures.

Finally, the present work should be read as a feasibility study based on immediate postoperative finite element models, which should be accompanied by experimental tests and clinical validation to effectively confirm the applicability of this material for LIF surgery.

## 5 Conclusion

This work supported brittle ceramics as analternative to standard material for PLIF and XLIF cages, ensuring effective primary stability and safely withstanding physiological loading conditions. Furthermore, the ceramic cage proved better than standard titanium in reducing the strains on the adjacent vertebrae; therefore, it may reduce the risk of subsidence. Future studies should focus on the design and optimization of a novel biodegradable ceramic interbody cage to further enhance its biomechanical performance.

## Author Contributions

**Davide Ninarello:** conceptualization, data curation, formal analysis, methodology, visualization, writing – original draft, writing – review and editing, validation, investigation. **Luigi La Barbera:** conceptualization, funding acquisition, investigation, methodology, project administration, resources, supervision, validation, visualization, writing – original draft, writing – review and editing.

## Funding

Davide Ninarello is supported by the Italian Ministry of University and Research through the project: NextGenerationEU program, Italia domani—Piano Nazionale di Ripresa e Resilienza, and GreenBone Ortho S.p.A (Faenza, Italy).

## Supporting information


**Figure S1:** Set‐ups for mechanical characterization of the bioceramic scaffold under axial compression (a), transverse compression (b) and axial torsion (c).
**Table S1:** Results of the mechanical experimental characterization of the bioceramic (b.Bone).

## Data Availability

All necessary data are fully disclosed within the paper and provided in detailed tables. Any editable data may be provided upon reasonable request to the corresponding author.
