# Supplementary material for: Are Ceramic Cages an Option for Lumbar Interbody Fusion? A Comparative Biomechanical Numerical Study
Source: JOR Spine. 2026 Sep 1;9(3):e70215. doi: 10.1002/jsp2.70215 (PMC13531705; doi:10.1002/jsp2.70215)
Supplement: Supplementary file 1 [file JSP2-9-e70215-s001.docx]

**Title:** Are ceramic cages an option for lumbar interbody fusion? A comparative biomechanical numerical study

**Authors:** Davide Ninarello^1^, Luigi La Barbera^1^

**Affiliations:** 1. Laboratory of Biological Structure Mechanics, Department of Chemistry, Materials and Chemical Engineering “Giulio Natta”, Politecnico di Milano, Milan, Italy

**Email Addresses:** davide.ninarello@polimi.it, luigi.labarbera@polimi.it

**ORCID IDs:**

**Davide Ninarello:** <https://orcid.org/0000-0002-8475-6694>

**Luigi La Barbera:** <http://orcid.org/0000-0002-9822-2116>

**Corresponding author:**	Davide Ninarello (davide.ninarello@polimi.it)

Laboratory of Biological Structure Mechanics, Department of Chemistry, Materials and Chemical Engineering “Giulio Natta”, Politecnico di Milano Piazza Leonardo da Vinci 32, 20133, Milan, Italy

**A – Mechanical characterization of the bioceramic material**

The methods and results presented in our previous work [23] are here resumed. The bioceramic material (b.Bone™) considered in the present work was previously mechanically characterized under different loading conditions (Fig. S1). A total of 29 samples were tested in static conditions under axial compression (n=13), axial torsion (n=10) and transverse compression (n=6). The terms “axial” and “transverse” are referring to directions along the main longitudinal porosity of the material or orthogonal to it, respectively.

The results of the experimental campaign are resumed in Table A1, highlighting the statistically significant difference between axial and transverse compressive mechanical properties. The material parameters obtained from this experimental analysis were used to calibrate a transversely isotropic material model assigned to the ceramic LIF cages.

**Fig. S1** Set-ups for mechanical characterization of the bioceramic scaffold under axial compression (a), transverse compression (b) and axial torsion (c). Local material reference system is represented for each loading configuration.

**Table S1** Results of the mechanical experimental characterization of the bioceramic (b.Bone™). Normally distributed data are presented as mean ± standard deviation, while non-normally distributed data are presented as median (minimum-maximum)

| **Mechanical Test** | **Elastic Modulus** | **Ultimate stress** | **Ultimate Strain** |
| --- | --- | --- | --- |
|  | *GPa* | *MPa* | *%* |
| Axial Compression | 4.65 *±* 2.25^*^ | 17.93 *±* 4.56^*^ | 0.43^*^(0.27-1.06) |
| Transverse Compression | 1.60 *±* 0.22^*^ | 3.37 *±* 1.02^*^ | 0.27^*^(0.17-0.31) |
| Axial Torsion | 1.39 *±* 0.57 | 2.62 *±* 0.62 | 0.19 *±* 0.06 |
| *: statistically significant difference between axial and transverse compression (p<0.01) | | | |

Among the 6 parameters characterizing the transversely isotropic constitutive model, E_zz_, E_xx_=E_yy_ and G_xz_=G_yz_ were estimated from respectively the experimental test of axial compression, transverse compression and axial torsion, from which ν_xz_=ν_yz_ were obtained according to the following equation:

$$\nu_{\mathrm{xz}}=\nu_{\mathrm{zx}}\frac{E_{xx}}{E_{\mathrm{zz}}}$$

where ν_zx_ is the Poisson’s ratio that defines the strain in the plane of isotropy resulting from stress normal to it, assumed equal to 0.27. Similarly, ν_xy_ was assumed equal to 0.27 and the parameter G_xy_ was obtained from:

$$G_{xy}=\frac{E_{yy}}{2*(1+ \nu_{\mathrm{xy}})}$$

The final compliance matrix assigned to the FE model is:

$$\boldsymbol{D}= \left[ \begin{matrix} \frac{1}{E_{x}} & -\frac{\nu_{yx}}{E_{y}} & -\frac{\nu_{zx}}{E_{z}} & 0 & 0 & 0 \\ -\frac{\nu_{xy}}{E_{x}} & \frac{1}{E_{y}} & -\frac{\nu_{zy}}{E_{z}} & 0 & 0 & 0 \\ -\frac{\nu_{xz}}{E_{x}} & -\frac{\nu_{yz}}{E_{y}} & \frac{1}{E_{z}} & 0 & 0 & 0 \\ 0 & 0 & 0 & \frac{1}{G_{xy}} & 0 & 0 \\ 0 & 0 & 0 & 0 & \frac{1}{G_{xz}} & 0 \\ 0 & 0 & 0 & 0 & 0 & \frac{1}{G_{yz}} \end{matrix} \right]=\left[ \begin{matrix} 0.63 & -0.17 & -0.06 & 0 & 0 & 0 \\ -0.17 & 0.63 & -0.06 & 0 & 0 & 0 \\ -0.06 & -0.06 & 0.22 & 0 & 0 & 0 \\ 0 & 0 & 0 & 1.59 & 0 & 0 \\ 0 & 0 & 0 & 0 & 0.72 & 0 \\ 0 & 0 & 0 & 0 & 0 & 0.72 \end{matrix} \right] GPa^{-1}$$
